# The improved Clinical Global Impression Scale (iCGI): development and validation in depression

**DOI:** 10.1186/1471-244X-7-7

**Published:** 2007-02-06

**Authors:** Alane Kadouri, Emmanuelle Corruble, Bruno Falissard

**Affiliations:** 1 Inserm, U669, Paris, France; 2 Univ Paris-Sud and Univ Paris Descartes, UMR-S0669, Paris, France; 3 AP-HP, Hôpital de Bicêtre, Service de Psychiatrie, Le Kremlin-Bicêtre, France; 4 AP-HP, Hôpital Paul Brousse, Département de santé publique, Villejuif, France

## Abstract

**Background:**

The Clinical Global Impression scale (CGI) is frequently used in medical care and clinical research because of its face validity and practicability. This study proposes to improve the reliability of the Clinical Global Impression (CGI) scale in depressive disorders by the use of a semi-standardized interview, a new response format, and a Delphi procedure.

**Methods:**

Thirty patients hospitalised for a major depressive episode were filmed at T1 (first week in hospital) and at T2 (2 weeks later) during a 5' specific interview. The Hamilton Depressive Rating Scale and the Symptom Check List were also rated. Eleven psychiatrists rated these videos using either the usual CGI response format or an improved response format, with or without a Delphi procedure.

**Results:**

The new response format slightly improved (but not significantly) the interrater agreement, the Delphi procedure did not. The best results were obtained when ratings by 4 independent raters were averaged. In this situation, intraclass correlation coefficients were about 0.9.

**Conclusion:**

The Clinical Global Impression is a useful approach in psychiatry since it apprehends patients in their entirety. This study shows that it is possible to quantify such impressions with a high level of interrater agreement.

## Background

The overall impression during an interview is a main element of psychiatric evaluation, but it is vague and difficult to operationalise. The Clinical Global Impression scale (CGI) is a classic instrument for making global assessments [1]. This scale yields three different measures: 1. Severity of illness (assessment of patient's current symptom severity, referred to here as CGIs), 2. Global improvement (comparison of patient's baseline condition with his/her current condition, referred to here as CGIi), 3. Efficacy index (comparison of patient's baseline condition with a ratio of current therapeutic benefit to severity of side effects). The CGI has been widely used in clinical research and especially in clinical trials concerning psychotropic treatments, for bipolar-disorder [2], anxiety [3] or schizophrenia [4]. Most often, the CGI scale is used with the same format or wording whatever the pathology under study. The CGI has nevertheless been more specifically adapted for bipolar disorder [2] and schizophrenia [4]. To our knowledge, there is, to date, no adaptation for depressive disorders.

The question of the validity of the CGI is still debated [5,6]. The scale is sensitive enough to differentiate respondents versus non responders in clinical trials in depression [7]. Its specificity is however disputed [8]. Cicchetti and Prusoff [9] followed 86 patients with a depressive episode for four months; the CGI showed poorer interrater reliability than the Hamilton Depressive Rating Scale (HDRS).

Many weaknesses could explain this possible lack of validity of the CGI: there is no specific interviewer guide available, and while most other symptoms scales have fairly clear and specific response options, the response format used in the CGI to assess change or severity of illness is more likely to be ambiguous (what is the definition of a patient who is "Severely ill"?).

This paper proposes a methodology that aims to improve the validity of CGI severity and improvement scales in the field of depressive disorders. Content validity will be improved by the design of a new response format. Interrater reliability will be improved by the drafting of a specific interviewer guide, a video recording of a specific 5-minute interview, and a multiple raters procedure using a short and modified Delphi procedure [10-12] to reach a consensus in ratings. The Delphi procedure requires the opinion of subjects each interviewed independently. The process is iterative: in a first round the subjects are invited to provide an opinion on a specific matter; in a second round they are invited again to provide their opinion, but with the opportunity to change it in view of the group's response; in a third round they are invited again to provide their opinion in view of the new group's response, etc. If an acceptable degree of consensus is obtained the process may cease.

The hypothesis was that both the new response formats proposed for the CGIs, and the restricted Delphi procedure, should improve the reliability of ratings in terms of interrater agreement.

## Methods

### Design of the "improved" CGI (iCGI)

#### Response format

The original response format for the CGIs is: 0 "not assessed", 1 "Normal, not at all ill", 2 "Borderline mentally ill", 3 "Mildly ill", 4 "Moderately ill", 5 "Markedly ill", 6 "Severely ill", 7 "Among the most extremely ill patients".

To improve the reliability of the severity scale (CGIs) the proposal is to back up the original response format with 13 case vignettes of patients (a vignette is a short written portraits, 2 vignettes are proposed for each answer ranging from 1 to 7 to take into account the heterogeneity of depressive symptoms). For instance, the response2 "Borderline mentally ill" is supported by the two vignettes: "The patient complains of periodic tiredness, unhappiness or loss of optimism, but this does not affect his/her relationships or job", or "Working life and family life are a little less pleasant for the patient. He/she describes moments of sadness and internal tension". The objective is not to directly compare the patient with the description, but to compare the impression of patient's level of severity with the impression of level of severity generated by the patients described in the portraits. In other words, with the usual CGIs response format the rater's standard for comparison is implicit and internal, while, with the case vignette, we propose an explicit and external standard for comparison.

Some elements of validation are required concerning this new response format. For this purpose, 6 psychiatrists were given the 12 case vignettes corresponding to responses 2 to 7 in a randomised order, and asked to arrange them in pairs and to order the pairs from the mildest to the greatest level of severity. Four of the experts responded in the expected order but two experts reversed the same two case vignettes (one of the two relating to response 2 "Borderline mentally ill" and one to response 3 "Mildly ill"). One of these case vignettes was then slightly modified, after which a new ranking by the two experts reached the consensus. The final set of case vignettes is presented in Table 1.

**Table 1:** Improved response format for the Clinical Global Impression severity scale in depression.

**Normal, not at all ill**
The patient has no symptoms to suggest depression
**Borderline mentally ill**
The patient complains of periodic tiredness, unhappiness or loss of optimism, but this does not affect his/her relationships or job.
Working life and family life are a little less pleasant for the patient. He/she describes moments of sadness and internal tension.
**Mildly ill**
The patient is tired, has difficulty taking initiatives or making an effort. Labile mood. At times, deterioration of professional performance.
The patient is tense and irritable. He/she has difficulty concentrating on daily tasks, although he/she mostly gets them done.
**Moderately ill**
The patient is sad and talks about waves of anxiety. His/her nights are restless. His/her professional life is taking the toll despite efforts to face up to it.
The patient has to fight against moments of despair. He/she is exhausted. His/her relationships are affected.
**Markedly ill**
The patient is listless, says he/she cries easily. He/she is eating irregularly, the face is thin. He/she complains of an impoverished emotional life, he/she can see no future.
The patient is no longer able to struggle against his/her sad mood. He/she describes a permanent state of internal tension. Everything is difficult to bear.
**Severely ill**
The patient is without reaction, permanently overwhelmed with his/her sad and painful mood. He/she is not eating.
The patient's face and utterance are devoid of affects. He/she has no plans, and says he/she is waiting to die.
**Among the most extremely ill patients**
The patient is cachectic, utterances are incoherent and centred on morbid themes. Distress is extreme.
The patient is prostrate, eyes averted. The face expresses painful tension. The interview is virtually impossible because of a refusal to communicate. The few utterances are delirious.

The original response format for the CGIi is: 0 "Not assessed", 1 "Very much improved", 2 "Much improved", 3 "Minimally improved", 4 "No change", 5 "Minimally worse", 6 "Much worse", 7 "Very much worse". This format corresponds to rating improvement on a 0–3 range. The new proposal is a 0–6 range for improvement, which is sometimes preferred in terms of sensitivity [13-15], it is presented in Table 2.

**Table 2:** Improved response format for the Clinical Global Impression improvement scale.

6 Ideal improvement
5 Very considerable improvement
4 Considerable improvement
3 Moderate improvement
2 Slight improvement
1 Very slight improvement
0 State unchanged.
-1 Very slight deterioration
-2 Slight deterioration.
-3 Moderate deterioration
-4 Considerable deterioration
-5 Very considerable deterioration
-6 Maximum deterioration

#### Interview

A clinical interview was specifically developed for the CGI in the field of depression. The objective of this interview is to obtain a 5-minute video where the material provided by the patient is expected to facilitate the formation of an "impression", an empathetic feeling about the patient's "essence" [16-19]. This interview is based on the voluminous literature that deals with the application of phenomenological concepts in psychiatry, phenomenology being a philosophical movement where the notions of impression and essence are central [16,18,19]. A recent review of the impact of phenomenology in north American psychiatric assessment has been proposed by Mona Gupta [20].

The clinical interview is close to day-to-day clinical practice, it is divided into three stages. The initial stage corresponds to the beginning of the interview. The first question is "Hello, how are you?". According to the nature of the response, the following strategies will be adopted.

- The patient expresses his/her inner emotions fluently: no intervention

- The patient encounters some difficulty talking about him/herself and remains on a superficial plane: the question may be rephrased in a more direct way: "Can you tell me what is going well and what isn't just now?". The objective is not necessarily for the patient to answer the questions, but rather for the person to talk and express him/herself about how things are at the moment.

The second stage corresponds to the main body of the interview. The objective is not to direct the patient towards particular areas but rather to make the interview as sensitive as possible. A few examples will help:

- The patient digresses: closed questions can be asked: "Could you be more explicit about this? Could you come back to that?

- The patient does not digress but gets lost in detail. One question may be: "What is the main point in what you said just now?"

- The patient answers, but is not explicit about his/her inner emotions. Some questions can be suggested to clarify: "What do you mean by this phrase?"

The third stage is the end of the clinical interview. Two questions can be asked at this point (if they have not been raised before): "How do you see the future? Do you sometimes think of death?". The final question can be "Can you say something else about yourself to help us to understand your mind?".

It is noteworthy that silences also play an important role. Silences arising from the patient (retardation, lack of insight) or silences arising from the interviewer (which may generate restlessness or irritation). From a technical point of view, the patient's head, chest and hands should be on the video so that non-verbal behaviours can be captured.

#### Procedure

Thirty patients hospitalised for a DSM IV major depressive episode [21] were included and filmed at T1 (first week in hospital) and at T2 (2 weeks later). The interview included the CGI clinical interview presented above and a semi-structured interview intended to score the HDRS. This last interview was not filmed and by the way not used for the CGI ratings. The SCL-90 was then completed by the patient. All the interviews were conducted by the same psychiatrist. Videos were sent to raters who were unaware of each other's identity. Exclusion criteria for patients were: age < 18 years, language difficulties, neurological pathologies.

### Sampling of raters

Eleven psychiatrists were asked to participate in the study as raters. They were selected on the basis of a wide variety of practice and characteristics (psychoanalysts or psycho-pharmacologists, university hospitals or psychiatric hospitals, young or experienced, etc.), but, deliberately, none had more than limited experience in the use of psychiatric scales. These selection criteria were decided on so that interrater agreement could not be suspected of being overestimated due to excessive homogeneity among the raters. The eleven raters were randomised into three groups. Three raters (group 1) were asked to rate the videos with the original response formats for the CGIs and CGIi. Four others (group 2) were asked to rate the videos with the new response formats for the CGIs and CGIi. The last four raters (group 3), were divided into two pairs (pair 1 and pair 2), each of these pairs worked with the new response formats of the CGIs and CGIi and with a restricted Delphi procedure. The scoring process for this was as follows: in a first round, each expert rates the CGIs and the CGIi. In a second round, each expert receives the assessment by the other expert, reviews the videos and again rates the CGIs and the CGIi. In a third round, the ratings obtained are averaged between experts.

These three groups were then used to assess and compare the reliability (in terms of interrater agreement) of the traditional response formats of CGIs and CGIi, the new response formats and the restricted Delphi procedure. It is also of a potential interest to assess the reliability of the average of the ratings by 4 independent clinicians. 4 ratings are proposed here since the restricted Delphi procedure also requires 4 evaluations (2 raters on 2 occasions): thus the workload of the two approaches is comparable. In practice, group 2 was therefore used to assess the reliability of the average of 4 ratings (see statistical section for details).

### Sampling of patients

Patients were hospitalised in Kremlin-Bicêtre university hospital psychiatric department, Paul Brousse university hospital psychiatric department and Clermont de l'Oise psychiatric hospital.

### Statistical methods

Interrater agreement is assessed using the intraclass correlation coefficient (ICC) as defined by the ratio of the inter-patient variance to the sum of the inter-patient variance, the interrater variance and the residual variance [22]. In group 1 three ratings are used for each patient. In group 2 four ratings are used. In group 3, two ratings are used, each derived from the mean of the last 2 ratings observed in each pair. It is noticeable that even if the intraclass correlation coefficients are obtained from different numbers of raters (i.e. three for group 1, four for group 2 and three for group 3), it is nevertheless possible to compare them statistically without any bias. However, in an ideal experimental design, 4 raters could have been preferred in group 1 so that a comparable precision of the estimated ICC would have been obtained in group 1 and in group 2.

Interrater agreement for the mean of 4 ratings derived from 4 independent psychiatrists can be estimated from group 2. In this situation, the intraclass correlation coefficient is obtained from the ratio of the inter-patient variance to the sum of the inter-patient variance, the interrater variance divided by 4 and the residual variance divided by 4 [23]. This formula shows that it is possible to estimate the intraclass correlation coefficient of a mean of k ratings even if fewer raters are available during the validation procedure.

A preliminary estimate of responsiveness is assessed by the effect size as defined by the ratio of the difference in raw scores between the two times of evaluation to the standard deviation of the score in the first time group [24].

Confidence intervals of intraclass coefficients (ICC) and effect sizes (ES) are obtained by a bootstrap procedure. Statistical tests of significance of ICC and ES are obtained using a bootstrap procedure which takes into account that the same population is used to compare these coefficients.

All statistical analyses were carried out on R 1.9 software and its "boot" and "psy" library [25].

### Ethics

Each patient signed an authorisation for the video and for the participation in the research. The protocol was approved by the "Comité d'évaluation éthique de l'inserm" (IRB0000388, FWA00005831).

## Results

Among the 30 patients included there were 25 females; the mean age was 49 years, with a standard-deviation of 12 years and a range of 27 to 80 years.

At T1, the HDRS, SCL90 and CGIs scores for group 1, group 2 and group 3 were highly correlated with a mean Pearson correlation coefficient of 0.74. More precisely, the Pearson correlation coefficient of the mean of CGIs scores for group 1, 2 and 3 and HDRS was equal to 0.81. The correlation of this mean of CGIs scores and SCL90 was equal to 0.62. The correlation between the HDRS and the SCL90 was equal to 0.69.

Table 3 presents the intraclass correlation coefficients which estimate the interrater reliability in groups 1, 2, 3 and 2' (the average of 4 ratings). All values are above 0.6, which corresponds to "good" to "excellent" agreement. All values for 2' (the average of 4 ratings) are above 0.75 which is considered to be "excellent" [26-28]. The hypotheses were that agreement in group 2 (new response format) would be more marked than agreement in group 1 (usual response format), and that agreement in group 3 (new response format and Delphi procedure) would be greater than agreement in group 2 (new response format only): data did not really support these hypotheses. If the level of agreement in group 2 appears systematically slightly superior to the level of agreement in group 1, this difference is however statistically significant only for the CGIs at T2. The Delphi procedure appears inefficient since the levels of agreement in group 3 are comparable with the levels of agreement in group 2. The level of agreement in group 2' (the average of 4 CGI scores obtained with the new response format) is higher than in group 2. These differences are inevitable from a statistical point of view and by the way cannot be tested, they are ranging from 0.23 to 0.14. It is also noticeable that the level of agreement in group 2' is systematically higher than in group 3 while the same amount of clinical workload is engaged. These differences in levels of agreement between group 2' and group 3 are significant at the 5% level for CGIs at T2, for the difference of CGIs between T2 and T1 and for CGIi.

**Table 3:** Intraclass correlation coefficient of CGI ratings and their 95% confidence intervals.

	CGIs1	95% C.I.	CGIs2	95% C.I.	CGIs1–2	95% C.I.	CGIi	95% C.I.
Group 1	0.64	[0.38, 0.77]	0.70	[0.53, 0.81]	0.64	[0.49, 0.77]	0.65	[0.39, 0.79]
Group 2	0.65	[0.40, 0.79]	0.80	[0.70, 0.87]	0.74	[0.54, 0.82]	0.74	[0.56, 0.84]
Group 3	0.86	[0.72, 0.93]	0.74	[0.57, 0.85]	0.69	[0.41, 0.84]	0.71	[0.53, 0.81]
Group 2'	0.88	[0.73, 0.94]	0.94	[0.90, 0.96]	0.91	[0.79, 0.95]	0.92	[0.83, 0.95]

CGIs measures severity at T1 (CGIs1) at T2 (CGIs2) and CGIi improvement between T2 and T1. CGIs1–2 measures the difference between the CGI severity scores measured at T2 and T1. Group 1 corresponds to 3 raters using the traditional CGI response format. Group 2 corresponds to 4 raters using the improved response format. Group 3 corresponds to a pair of ratings derived from a restricted Delphi procedure (each rating is obtained from 2 clinicians) and using the improved response format. Group 2' is Group 2, but the intraclass correlation coefficient computed here estimates the reliability of the average of 4 ratings derived from independent raters.

These results suggest to compare the level of agreement between the average of 4 CGI scores obtained with the new response format (group 2') and the average of 4 CGI scores obtained with the usual response format (which could correspond to a group 1'). For group 1', we have then: at T1, ICC = 0.88 (0.88 also for group 2'); at T2, ICC = 0.90 (0.94 for group 2'); for the difference between T2 and T1, ICC = 0.88 (0.91 for group 2') and for the CGIi, ICC = 0.88 (0.92 for group 2'). Here again, these differences are statistically significant only at T2.

The lack of efficiency of the Delphi procedure may be explained in part from the fact that the two raters in pair 1 are rather divergent in their first rating and they maintain their position in their second rating (data not shown).

Table 4 presents effect sizes computed from variations in scores between T1 and T2 using the Cohen d formula [24]. Table 4 shows that the effect size is larger in group 2' (ES = 1.02) than with the HDRS scale (ES = 0.61) or with the SCL90 scale (ES = 0.54). This difference in effect size is significant at the 5% level.

**Table 4:** Effect sizes computed from 30 depressed patients evaluated at two times (baseline and after two weeks in hospital, see table 3 for definition of Group 1, 2, 3 and 2')

	Group 1	Group 2	Group 3	Group 2'	HDRS	SCL
Effect size	0.93	0.93	0.76	1.02	0.64	0.51
95% C.I.	[0.58, 1.71]	[0.54, 1.60]	[0.43, 1.26]	[0.55, 1.75]	[0.33, 1.06]	[0.26, 0.89]

## Discussion

This paper suggests that, in the field of depression, a Clinical Global Impression, when elaborated with an appropriate methodology, can be operationalised into a reliable measure. This "improved" CGI (iCGI) entails a specific 5' video-recorded clinical interview, a new response format and a group of 4 independent clinicians to score the videos. The average of the 4 scores is considered as the final measurement.

Data tends to show that, in depression, the group of independent clinicians do not need to conduct a Delphi procedure: if clinicians are too far from each other in the first round, it appears that they do not move towards consensus in the next round. If the Delphi method can establish consensus for diagnosis in the carpal tunnel syndrome [11], it does not seem to work for the assessment of intensity of depression. Therefore, a group of 4 clinicians whose evaluations are averaged will be preferred to a group of 2 clinicians using a two-round Delphi procedure.

A response format specific to the field of depression has been developed. Its use results in a slight increase in interrater reliability which is nevertheless statistically significant only at T2. It can be suggested then to use the case vignettes for the iCGI severity scale and the -6/0/6 response format instead of the usual -3/0/3 one for the iCGI improvement scale. This last point was not evident since there is an old controversy about the optimal number of response categories in rating scales [13-15]. It is remarkable that this limited benefit induced by the new response format makes it conceivable to use the iCGI with the usual response format of the CGIs and CGIi. The iCGI could be used by the way in other fields than depression.

It could be objected that there was no use of a structured interview to assess the diagnosis of DSM IV major depressive episode. But the iCGI is designed to deal with a broad spectrum of depressive disorders.

It could also be objected that the iCGI is much more demanding than a classic HDRS. Our experience does not confirm this. The 5-minute video-recording is not a real burden for the clinician: the use of a high-resolution webcam and a microphone plugged into a computer make things quite simple. For a given evaluation, an iCGI requires about 30 minutes: 5 minutes for the interview, 5 minutes × 4 for the ratings plus 5 minutes "down-time". Moreover, the use of video makes it possible to implement quality control procedures, and the short duration of the interview makes it possible to replicate assessment in a given day at different times, in order to reduce the error component due to nycthemeral fluctuations. However, there is no doubt that such a procedure will be very difficult to implement in a population based study.

An encouraging but preliminary result resides in the effect sizes. The iCGI sensitivity to change which is demonstrated by the effect sizes is significantly and substantially larger than the HDRS. This can be translated in terms of numbers of subjects to include in a study, for a given power and to show a given difference. A protocol that would require 300 subjects with the HDRS, would require only 119 patients with the iCGI. Of course, these results should be confirmed, for example because the effect sizes are computed on T1/T2 measurements and not on treatment groups. Furthermore, this result makes sense only if measurement error is the main source of error. In the field of depression, circadian fluctuations of symptoms are important and may be an important source of variance. A repetition of iCGI evaluations within or across days could then be necessary in order to improve substantially the power of studies.

Of course, one of the most important drawback often opposed to the CGI, its lack of specificity [8], may also be opposed to the iCGI. Indeed, when used in depressed patients, the CGI scales capture potentially, in unknown proportions, many characteristics that are not strictly related to the depressive symptomatology. Personality traits, comorbidity, level of dangerousness, level of functioning are among these characteristics. Two arguments may be proposed to soften this drawback. First, the new response format of the iCGIs is likely to help the clinician to focus on the depressive dimension. Second, when assessing the efficiency and even the efficacy of a drug or more generally of a treatment, it is actually useful to have an outcome measurement that does not concentrate only on the academic disorder under study, but rather on the patient himself, grasped in his/her entirety.

## Conclusion

The iCGI could be an interesting tool in the global evaluation of psychiatric patients. It is studied here in the field of depression. Its clinical relevance, interrater reliability, sensitivity to change and possibility to implement quality control processes make it of a potential use in many areas of clinical research in psychiatry.

## Abbreviations

CGI (Clinical Global Impression scale). T1 (first week in hospital). T2 (2 weeks later). HDRS (Hamilton Depressive Rating Scale). SCL90 (Symptoms Check list 90). CGIs (Clinical Global Impression severity scale). CGIi (Clinical Global Impression improvement scale). iCGI (Improved Clinical Global Impression Scale). ICC (intraclass correlation coefficient). ES (effect size).

## Competing interests

The author(s) declare that they have no competing interests.

## Authors' contributions

BF, AK and EC have all participated to the design of the study protocol. BF and AK analysed the data under the statistical expertise of BF. AK videotaped the patients. All authors read and approved the final manuscript.

## Pre-publication history

The pre-publication history for this paper can be accessed here:


